# Aptamer technology: a new approach to treat lymphoma?

**DOI:** 10.1097/BS9.0000000000000033

**Published:** 2020-01

**Authors:** Youli Zu

**Affiliations:** Department of Pathology and Genomic Medicine, Houston Methodist Hospital, Houston, Texas, USA

**Keywords:** Immunotherapy, Lymphoma, Oligonucleotide aptamer, Precision therapy, Target therapy

## Abstract

Oligonucleotide aptamers are a class of small-molecule ligands. Functionally similar to protein antibodies, aptamers can specifically bind to their targets with high affinity. Biomedical studies have revealed the potential clinical value of aptamer technology for disease diagnosis and targeted therapy. Lymphoma is a group of cancers originating from the lymphatic system. Currently, chemotherapy is the primary treatment for lymphoma, although it may cause serious side effects in patients due to lack of target specificity. Here, we selectively discuss the recent development of potential applications of aptamer technology for precision lymphoma therapy, which are able to not only achieve high therapeutic efficacy but also do not cause off-target side effects.

## 1. INTRODUCTION

Aptamers are a new class of small-molecule ligands composed of short, single-stranded RNA or DNA.^1,2^ Target-specific aptamers can be developed through Systematic Evolution of Ligands by EXponential enrichment (SELEX) technology. In contrast to conventional nucleic acid molecular probes, aptamers precisely interact with their targets through structural recognition, a process similar to that of an antigen-antibody reaction. Because oligonucleotide aptamers are chemically synthesized and can specifically bind to their targets with high affinity, they are also referred to as “chemical antibodies.”^3–10^ Notably, due to their unique physical and chemical properties, aptamers offer several advantages over protein antibodies in biomedical research and clinical applications. Oligonucleotide aptamers (i) can be chemically synthesized at low production costs; (ii) can be easily modified and conjugated with a variety of functional moieties for biomedical and clinical use; (iii) theoretically are not immunogenic in vivo due to their short oligonucleotide nature; and (iv) have the capacity for efficient tissue penetration and fast cell binding.^8,11,12^ Therefore, as molecular recognition probes, aptamers have been widely studied to develop new approaches for disease diagnosis and target therapy.

Lymphoma is a group of cancers originating from the lymphatic system. Currently, chemotherapy is the primary treatment for lymphoma. Because chemotherapy is not lymphoma cell- or oncogene-specific, it may cause serious side effects and even increase the risk of a second malignancy in patients.^13–16^ To address these clinical challenges, new target therapeutic approaches using aptamer technology have been reported.^17–19^ Recent studies showed that these new approaches can enhance therapy efficacy and also reduce off-target side effects, helping to achieve precision medicine.

## 2. POTENTIAL APPROACHES OF APTAMER TECHNOLOGY FOR PRECISION LYMPHOMA THERAPY

### 2.1. Aptamer treatment to prime lymphoma cells for chemotherapy

**Figure 1.**
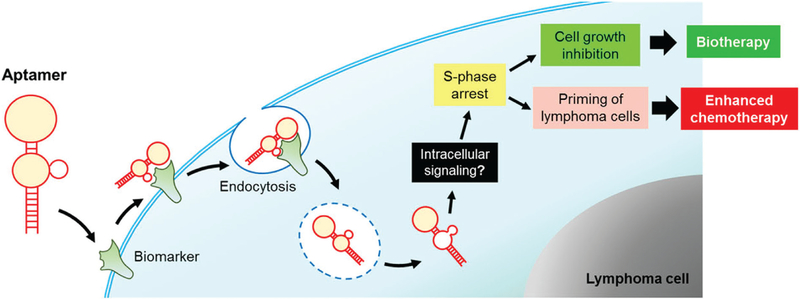
Scheme of aptamer-mediated biotherapy and enhanced chemotherapy for precision lymphoma therapy. Synthetic aptamers specifically bind to lymphoma cells and subsequently trigger endocytosis in the targeted cells. Although the exact underlying molecular mechanism is unknown, internalized aptamers regulate cellular signaling pathway(s) and then induce S-phase arrest of lymphoma cells, achieving biotherapy by controlling cellular biological functions without the use of toxic agents. In addition, the induced S-phase arrest by aptamer pretreatment is able to prime lymphoma cells for chemotherapies such as cytarabine, which selectively kills lymphoma cells at S-phase. The combination of aptamer-mediated biotherapy and cytarabine chemotherapy can achieve a synergistic effect on targeted cells, opening new avenues for precision lymphoma therapy.

To target lymphoma cells, we have conducted cell-based SELEX experiments and identified an ssDNA aptamer specifically targeting Maver-1 cells (a cell line of mantle cell lymphoma).^20^ Synthetic aptamers were able to specifically bind Maver-1 lymphoma cells with high affinity and subsequently triggered endocytosis of aptamers into the targeted cells. Interestingly, cellular function studies revealed that exposure of Maver-1 cells to aptamers induced S-phase arrest of 40% of the targeted cells (versus 18% baseline). Further investigation demonstrated that aptamer internalization into targeted cells is a prerequisite for S-phase arrest of Maver-1 cells. Findings suggested that aptamer treatment was able to regulate the cell growth cycle and thus control lymphoma cell growth through an unknown cellular signaling mechanism. More importantly, this aptamer-induced S-phase arrest also provided an opportunity to enhance chemotherapy efficacy, particularly by chemotherapeutic agents that selectively kill lymphoma cells at S-phase, such as cytarabine. As expected, in vitro studies indicated that pretreatment by aptamers significantly primed Maver-1 cells for subsequent cytarabine chemotherapy, achieving a synergistic killing effect by reaching cell death rates as high as 61% (versus 13% or 14% induced by aptamer pretreatment alone or cytarabine chemotherapy alone, respectively). This study demonstrated that aptamers do not only act as molecular ligands but can also function as a therapeutic agent to prime lymphoma cells by biologically regulating the cell growth cycle, resulting in biotherapy. Logical combinations of aptamer technology and different therapeutic modalities may open new avenues for precision lymphoma therapy (Fig. 1).

### 2.2. Aptamer nanomedicine for both lymphoma cell-selective chemotherapy and oncogene-specific gene therapy.

To eliminate adverse toxicity, antibody-drug conjugates have been used clinically for cell-selective chemotherapy. Anaplastic large cell lymphoma (ALCL) is the most common aggressive T-cell lymphoma in children and young adults.^21^ The lymphoma cells are characterized by high-level expression of the surface CD30 receptor, which is a distinct biomarker for diagnosis of the disease and target therapy.^22,23^ A CD30 antibody-drug conjugate (brentuximab vedotin) has been approved to treat refractory ALCL, although production of antibody-drug conjugates is labor-consuming and costly.^24,25^ In contrast, aptamer-drug conjugates can be easily produced through covalently or noncovalently conjugating synthetic aptamer sequences directly with therapeutic agents such as doxorubicin to treat lymphomas. Additionally, aberrant expression of the anaplastic lymphoma kinase (ALK) oncogene has been demonstrated to be a key pathogenic factor for ALCL development.^26,27^ Molecular studies revealed that the silencing of the ALK oncogene by small interfering RNA (siRNA) methods inhibited lymphoma growth.^28,29^ However, the clinical utility of siRNA therapy is limited mainly by ineffective delivery to cells of interest due to lack of cell specificity. Interestingly, because both siRNA and aptamers are synthetic oligonucleotides, siRNAs can be covalently or noncovalently conjugated with aptamers to form aptamer-siRNA chimeras for cell-specific gene therapy.

**Figure 2.**
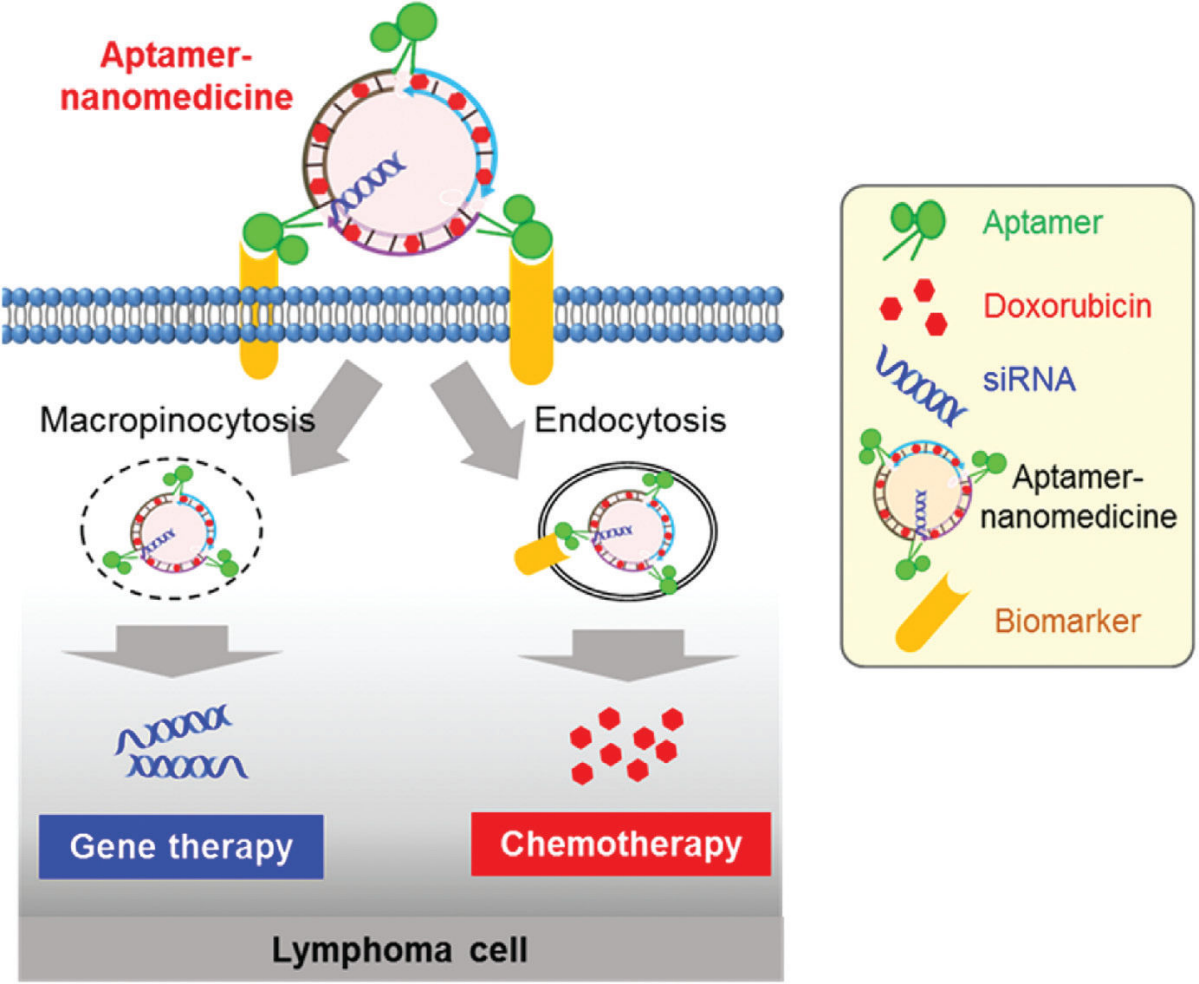
Scheme of aptamer nanomedicine for combined cell-selective chemotherapy and oncogene-specific gene therapy. The aptamer nanomedicine is formulated through programmatically structural self-assembly of three complementary oligonucleotides and a subsequent drug self-loading process. The formed aptamer nanomedicine is composed of three copies of aptamer sequences to target lymphoma cells and two different therapeutic modalities for cell-selective chemotherapy by doxorubicin drug payload and oncogene-specific gene therapy by the carried siRNA molecule, respectively. Post-systemic administration, the aptamer nanomedicine specifically targets surface biomarkers on lymphoma cells and concurrently performs chemotherapy to kill lymphoma cells via intracellular delivery and release of drug payload through aptamer-mediated cell endocytosis and gene therapy to silence the ALK oncogene and induce cell growth arrest via intracellular delivery of siRNA through aptamer-mediated cell macropinocytosis. By combining different therapeutic mechanisms and acting through different cellular signaling pathways, aptamer nanomedicine will have high therapeutic efficacy and no off-target toxicity, thereby achieving precision lymphoma therapy.

In order to combine chemotherapy and gene therapy designed for ALCL, a cell-selective and oncogene-specific aptamer nanomedicine was investigated, making use of aptamer technology. A nanostructure was initially formulated through programmatic self-assembly of three synthetic complementary oligonucleotides containing aptamer sequences specific for ALCL cells (K299 cell line) and a siRNA sequence specific for the cellular ALK oncogene. Subsequently, the aptamer nanomedicine was generated by a drug self-loading process via intercalation of doxorubicin into the formed nanostructures. Physical, chemical, and biological properties of the formed aptamer nanomedicine were characterized.^30^ As expected, functional studies showed that under aptamer guidance, the aptamer nanomedicine binds and then internalizes into targeted ALCL cells. Intracellular delivery of aptamer nanomedicine via endocytosis triggers rapid release of doxorubicin drug payload within the targeted cells for cell-selective chemotherapy. In addition, intracellular delivery of the carried siRNA via macropinocytosis specifically induced cellular ALK oncogene silencing. Concurrently functioning through different therapeutic mechanisms results in additional and synergistic inhibitory effects on ALCL cells (Fig. 2). Furthermore, xenograft animal model studies revealed that upon systemic administration, the aptamer nanomedicine specifically targets and selectively accumulates in the ALCL tumor, but it does not react with off-target tumor in the same mouse. Importantly, the aptamer nanomedicine treatment not only induces higher inhibition in lymphoma tumors but also causes fewer side effects in treated mice as compared to treatment with an equal amount of free doxorubicin. Moreover, aptamer nanomedicine treatment markedly improves the survival rate of treated mice, providing a new method for precision lymphoma therapy. Finally, given that aptamer nanomedicine is composed of biocompatible and biodegradable materials and can be easily produced through programmed design and self-assembly process, it will be highly transformative for clinical use. Notably, the aptamer nanomedicine is a universal platform and can be used to treat different types of cancers by simply replacing the aptamer sequence specific for cells of interest and the siRNA sequence for the underlying pathogenic oncogene.

### 2.3. Aptamer-engineered natural killer cells for cell-specific immunotherapy

Chimeric antigen receptor (CAR)-T cell technology has been recently approved for cancer immunotherapy and has shown highly promising results.^31^ CD30-specific CAR-T cells are also under investigation to treat lymphomas, including ALCL.^32^ However, CAR-T cell therapy has the potential to cause severe cytokine release storms and lethal neurotoxicity.^33,34^ In contrast to T cells, the safety of autologous, allogeneic, and cultured natural killer (NK) cells for adaptive immunotherapy has been demonstrated.^35^ More interestingly, NK cells are able to attack cancer cells without prior sensitization or clonal expansion and can induce the death of targeted cancer cells through exocytosis of cytotoxic granules.^36^ These unique inherent properties confer high value to NK cells in adaptive cancer immunotherapy. However, due to a lack of cell-specific receptors, NK cells cannot selectively target cancer cells, thus diminishing their immunotherapeutic potential. To render NK cells with target specificity, CAR-NK technology has emerged and its efficacy to treat lymphoma is currently under investigation.^37^ Producing CAR-NK cells, however, is labor-intensive and has inherent risks for patients, such as transgene insertional mutagenesis due to genetic manipulation and the viral material involvement required for CAR-NK cell production.

**Figure 3.**
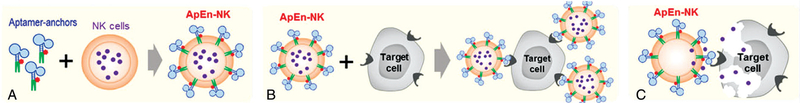
Schemes of aptamer-engineered NK (ApEn-NK) cells for lymphoma-specific immunotherapy. (A) Production of ApEn-NK cells. First, an aptamer-anchor structure was synthesized by using the aptamer sequence specific for ALCL cells. The ApEn-NK cells were then produced via simple incubation of naïve NK cells with aptamer-anchor structures, which are rapidly engineered on the NK cell surface through a programmatic anchoring reaction. (B) Specific lymphoma cell binding by ApEn-NK cells. Under surface-anchored aptamer guidance, the ApEn-NK cells specifically bind to biomarkers on lymphoma cells and form cell clusters with targeted cells. (C) Specific lymphoma cell killing by ApEn-NK cells. The aptamer-mediated cell binding will result in enhanced killing of targeted cells by ApEn-NK cells, achieving lymphoma-specific immunotherapy without side effects on off-target cells.

To develop a simple and safe immunotherapy approach, we recently reported an aptamer-engineered NK (ApEn-NK) cell method by taking advantages of aptamer technology.^38^ As shown in Figure 3A, a unique aptamer-anchor structure was first chemically synthesized using an aptamer sequence specific for ALCL cells. The ApEn-NK cells were then produced via rapid incubation of NK cells with aptamer-anchor structures that can be rapidly engineered on the NK cell surface through a programmatic anchoring reaction. It is expected that once equipped with surface-anchored aptamer sequences, the formed ApEn-NK cells will become ALCL cell-specific. Under aptamer guidance, the ApEn-NK cells will specifically bind with lymphoma cells, thus resulting in the selective killing of targeted cells with high efficacy (Fig. 3B and C). The in vitro studies revealed that in cell mixture, ApEn-NK cells specifically bound to and thus formed significantly more cell clusters with lymphoma cells than that observed in the cell mixture of lymphoma and parental NK cells under the same conditions. Subsequent cell killing assays demonstrated that ApEn-NK cells induced significant increases in apoptosis and the death of target lymphoma cells in cell mixtures, as compared to the baseline effect of parental NK cells in cell mixtures containing the same lymphoma cells. In addition, for preclinical validation, primary human naïve NK cells were isolated from peripheral blood of healthy donors and expended by in vitro culture. Similarly, functional studies showed that the ApEn-NK cells derived from primary human NK cells had the capacity for specific binding to lymphoma cells and enhanced killing of targeted cells in cell mixtures. Notably, because the production process does not involve genomic introduction/alteration or the usage of viral materials, the ApEn-NK cells are highly biocompatible and risk-free for clinical use. Taken together, this study validated a simple method to render naïve NK cells with target specificity and presents a new approach for cell-specific lymphoma immunotherapy by ApEn-NK cells, although the clinical value of this technology needs to be further evaluated.

## 3. CONCLUSION AND DISCUSSION

As chemical antibodies, oligonucleotide aptamers can be easily conjugated with different functional molecules per clinical needs for disease diagnosis and treatment. To demonstrate the clinical application capacity of aptamer technology, this article selectively discussed three potential approaches for precision lymphoma therapy. These studies indicate that logically combining aptamer technology with different therapeutic modalities will allow us to develop new personalized and/or precision therapeutic approaches that have high therapeutic efficacy for lymphoma and low off-target adverse effects in patients. To accelerate the translation of aptamer-mediated clinical applications from bench-side to bedside, close collaboration among scientists, chemists, and clinicians is indispensable. Although some technical challenges remain to be overcome, as more preclinical and clinical studies are conducted, aptamer technology can become a powerful tool in the near future for precision therapy to treat lymphoma, as well as other diseases.
